# Burden of tuberculosis in Xinjiang between 2011 and 2015: A surveillance data-based study

**DOI:** 10.1371/journal.pone.0187592

**Published:** 2017-11-09

**Authors:** Xiangyan He, Mingqin Cao, Tanmay Mahapatra, Xiangpin Du, Sanchita Mahapatra, Qifeng Li, Lei Feng, Songyuan Tang, Zhen Zhao, Jinbao Liu, Weiming Tang

**Affiliations:** 1 School of Public Health, Xinjiang Medical University, Urumqi, China; 2 The People’s Hospital of Xinjiang Uyghur Autonomous Region, Urumqi, China; 3 University of California Los Angeles, Los Angeles, California, United States of America; 4 Xinjiang Uyghur Autonomous Region Center for Disease Prevention and Control, Urumqi, China; 5 Xinjiang Institute of Pediatrics, Urumqi, China; 6 School of Public Health, Kunming Medical University, Kunming, China; 7 University of North Carolina Project-China, Guangzhou, China; 8 Dermatology Hospital, Southern Medical University, Guangzhou, China; 9 Guangdong ^2^Provincial Dermatology Hospital, Guangzhou, China; Temple University School of Medicine, UNITED STATES

## Abstract

**Background:**

Despite the reduction in reported incidence of tuberculosis globally, the burden of pulmonary tuberculosis (PTB) remains high in low- and middle- income countries, including China. The current study aims to evaluate the distribution and trend of PTB incidence in Xinjiang, the region with the highest PTB burden in China.

**Methods:**

We identified all confirmed PTB case records reported to the Chinese TB Information Management System (TBIMS) between 2011 and 2015. We analyzed these records to measure the annual incidence of reported smear-positive PTB cases in Xinjiang and its trend over time. We also analyzed incidence by gender, residential area, and region. Spatial analysis was used to describe the inter-regional disparity of the disease burden during the study period.

**Results:**

We identified 212,216 smear-positive PTB cases between 2011 and 2015. The reported incidence increased from 180.8 cases in 2011 to 195.8 cases in 2015 per 100,000 population. The southern region of Xinjiang had the highest disease burden (257.8/100,000 in 2011 and 312.7/100,000 in 2015). More than 60% cases occurred in persons >45 years, and 76% of cases lived in rural areas.

**Conclusion:**

To reach the goal of elimination and control of TB, more comprehensive STOP TB strategies should be implemented in Xinjiang. Residents in the southern region and rural areas of Xinjiang require particular attention.

## Introduction

Despite major achievements in tuberculosis (TB) prevention and control [1, 2], TB remains a major global public health issue, especially in the low- and middle- income countries. [3, 4]. In China, although TB prevalence has decreased by 50% over the last two decades [5], it remains alarmingly high in resource-limited settings. In several regions of western China, although the prevalence of smear-positive TB decreased by 25% between 1990 and 2010, the prevalence of bacteriologically confirmed pulmonary TB (PTB) increased by 17.8% [6].

The Xinjiang Uighur Autonomous Region is a resource-poor territory of western China with a population of 20 million people belonging to thirteen different ethnic groups [7]. Ethnic diversity and resulting conflict [7] coupled with stringent socio-cultural norms and religious practices [8] may have contributed to the limited development of this region as a whole [9]. As a result, the roll-out of a comprehensive TB control program was halted for years and only expanded to the whole region after 2000 [10, 11]. Consequently, the burden of TB in Xinjiang has remained one of the highest among all provinces in China during the last two decades. The reported incidence of smear-positive PTB was estimated to be as high as 231 and 170 per 100,000 people during 2000 and 2010, respectively [6, 12]. Although surveillance data showed a slight decrease in the reported incidence of smear-positive TB[6, 12], there was a simultaneous, conflicting increase in the number of bacteriologically confirmed cases [6]. Further, the majority of the studies conducted among TB patients residing in Xinjiang are burdened by small sample sizes and scarcity of local data [5, 6]. The Xinjiang TB epidemic thus remains grossly understudied [13].

The goal of this study was to develop an updated, valid estimate for the reported incidence of smear-positive TB in Xinjiang using a record-based analysis through data collected from the Chinese TB Information Management System (TBIMS). In addition, we sought to determine the distribution and variation of TB cases across time, geographic areas, and socio-demographic variables.

## Methods

### Data collection

We collected reported TB cases from 2011–2015 from the Chinese TB Information Management System (TBIMS, http://www.phsciencedata.cn/Share/index.js). Information regarding TBIMS has been described elsewhere in detail [14]. In brief, TBIMS is a registry, containing real-time information of TB cases reported from all provinces in China. Since 2011, TBIMS has been structured into three case-definition-based databases containing information on pulmonary, extra-pulmonary and drug-resistant (presumptive diagnosis) TB cases.

Each PTB case is diagnosed based on the case definitions per WHO guidelines and ICD-10 [15]. All PTB cases in mainland China are recorded in the PTB database of TBIMS within 24 hours of diagnosis. The diagnosis of PTB mainly depends on results of laboratory examination (sputum smear positivity), combined with chest imaging, personal history, clinical manifestations and necessary auxiliary examinations.

For the present study, we collected all cases recorded in the PTB database that were smear positive. We also collected information on reporting time, patient ID, address, age, gender, race, medical history, laboratory testing results, chest imaging results and clinical manifestations. To ensure the quality of the data, we only included cases that reported a set of specified key variables. [14]

### Data management

To protect confidentiality, all personal identification information was removed from the database before data analysis.

To confirm local residency and to exclude migrant populations, we only included cases who had been residing in their current household for at least last six months. We categorized the study population into five age-groups: 0–15, 16–30, 31–45, 46–60 and >60 years. Depending on the recorded address, the patients were classified into urban and rural residents. Residential areas were classified as southern, northern or eastern regions of Xinjiang and sub-classified into counties.

The population of Xinjiang and each of its cities were determined from the Statistical Yearbook of Xinjiang Uygur Autonomous Region and the Social Development Statistical Communique of Xinjiang Uygur Autonomous Region.

### Data analysis

We used descriptive analysis to examine the distribution of the demographic characteristics among the participants. We calculated the annual incidence of smear-positive PTB in Xinjiang by dividing the number of smear-positive PTB cases identified per year by the total population size. We also calculated the PTB incidence by region, gender and residential area (urban and rural) and evaluated trends in the reported incidence by examining patterns in crude OR.

ArcGIS 10.2 software (ESRI Inc., Redlands, CA, USA) was used for spatial analysis, to describe the inter-regional disparity of the disease burden during the study period.

### Ethics statement

The study protocol, contents, and procedures were reviewed and approved by the Institutional Review Board of the Xinjiang Uygur Autonomous Region CDC. The study was based on case records only and did not requre informed consent. The funders of the study had no role in study design, data collection, data analysis, data interpretation, or writing of the report. The Xinjiang CDC operated under the general guidance of the Xinjiang Uyghur Autonomous Region Health Bureau and was responsible for the survey design and field investigation.

## Results

### Demographics

Between 2011 and 2015, a total of 212,216 smears positive PTB cases were reported to TBIMS and included in the current study. The number of cases reported annually increased from 37,954 in 2011 to 46,205 in 2015. Among the included cases, 52.6% were male, 4.6% were aged ≤15 years, and 62.4% were aged >45 years (34.1% aged >60 years). 76.0% of the cases lived in rural areas of Xinjiang. The majority of the cases (71.8%) were reported from the southern region of Xinjiang Uygur Autonomous Region, whereas the eastern region reported only 2.9%. Also, 2.0% of the cases were reported from four counties directly managed by the province.

Overall, the gender and age distribution of the reported cases remained unaltered across the study period of five years. However, the proportion of cases reported from rural areas increased from 71.4% in 2011 to 77.6% in 2015 (*P* for trend <0.001). In addition, the proportion of cases reported from the southern region also increased from 66.4% in 2011 to 73.8% in 2015 (*P* for trend <0.001) (Table 1).

**Table 1:** Distribution of the yearly reported pulmonary tuberculosis cases in Xinjiang Uygur Autonomous Region, China, 2011–2015.

	2011		2012		2013		2014		2015		Overall	
	No.	%	No.	%	No.	%	No.	%	No.	%	No.	%
**Overall**	37954	100.0	42561	100.0	42309	100.0	43187	100.0	46205	100.0	212216	100.0
**Gender**												
*Male*	20258	50.7	22721	53.4	22259	52.6	22512	52.1	23792	51.5	111542	52.6
*Female*	17696	44.3	19840	46.6	20050	47.4	20675	47.9	22413	48.5	100674	47.4
**Age group (Years)**											0	
*0–15*	2114	5.3	1976	4.6	1923	4.5	1892	4.4	1830	4.0	9735	4.6
*15–30*	7061	17.7	7200	16.9	7119	16.8	6818	15.8	6732	14.6	34930	16.5
*30–45*	6265	15.7	7371	17.3	7009	16.6	7246	16.8	7223	15.6	35114	16.5
*45–60*	10110	25.3	12095	28.4	12199	28.8	12428	28.8	13229	28.6	60061	28.3
*>60*	12404	31.0	13919	32.7	14059	33.2	14803	34.3	17191	37.2	72376	34.1
**Residential area**												
*Urban*	9435	23.6	10293	24.2	10452	24.7	10493	24.3	10362	22.4	51035	24.0
*Rural*	28519	71.4	32268	75.8	31857	75.3	32694	75.7	35843	77.6	161181	76.0
**Region**												
*Northwest region*	9365	23.4	9727	22.9	10032	23.7	10192	23.6	10209	22.1	49525	23.3
*Southern region*	26525	66.4	30592	71.9	30104	71.2	30991	71.8	34080	73.8	152292	71.8
*Eastern region*	1235	3.1	1314	3.1	1257	3.0	1221	2.8	1149	2.5	6176	2.9
*Counties Administrated by Province*	829	2.1	928	2.2	916	3.0	783	1.8	767	1.7	4223	2.0

### Population reported incidence

Within the study period, the overall population level incidence of reported PTB increased over time, from 180.8 cases per 100,000 people (95% CI = 179.2–182.7) in 2011 to 195.8 cases per 100,000 people (95% CI = 194.0–197.6) in 2015. The annual incidence of reported PTB among males was slightly higher than females (not significant), except in 2015. Reported incidence of PTB among females increased from 163.9 cases per 100,000 people (95% CI = 161.4–166.3) in 2011 to 197.9 cases per 100,000 people (95% CI = 195.3–200.5) in 2015 (Table 2). Detailed case numbers, population sizes, and demographics are provided in S1 File.

**Table 2:** Population level occurrence and trend of pulmonary tuberculosis in Xinjiang Uygur Autonomous Region, China, 2011–2015.

	2011		2012		2013		2014		2015		Crude OR*
	Cases (/100,000)	95% CI	Cases (/100,000)	95% CI	Cases (/100,000)	95% CI	Cases (/100,000)	95% CI	Cases (/100,000)	95% CI	
**Overall**	180.8	179.2–182.7	190.6	188.8–192.4	186.9	185.1–188.6	187.9	186.1–189.7	195.8	194.0–197.6	1.08(1.07,1.09)
**Gender**											
*Male*	179.5	177.0–181.9	195.7	193.2–198.2	189.0	186.6–191.5	193.3	190.8–195.9	193.9	191.4–196.3	1.08(1.06,1.10)
*Female*	163.9	161.4–166.3	185.1	182–187.7.6	184.5	181.9–187.0	182.3	179.8–184.8	197.9	195.3–200.5	1.21(1.19,1.22)
**Stratum**											
*Urban*	98.1	96.1–100.1	104.8	102.8–106.8	103.8	101.8–106.8	99.1	87.2–101.0	93.0	91.2–94.8	0.95(0.93,0.98)
*Rural*	228.7	226.0–231.4	258.0	255.2–260.8	253.4	250.6–256.1	263.8	260.9–266.6	287.8	284.8–290.8	1.26(1.23,1.29)
**Region**											
*Northwestern*	99.7	97.6–101.7	102.0	100.0–104.0	103.0	101.0–105.0	104.0	102.0–106.0	100.7	98.7–102.6	1.01(0.99,1.02)
*Southern region*	257.8	254.7–260.9	296.5	293.2–299.8	289.7	286.4–293.0	290.9	287.7–294.2	312.7	309.4–316.0	1.21(1.19,1.23)
*Eastern region*	102.2	96.5–107.9	107.1	107.1–112.9	101.3	95.7–106.8	97.7	92.2–103.2	90.2	85.0–95.4	0.88(0.79,0.99)
*Counties Administrated by Province*	69.6	64.9–74.4	74.5	69.7–79.3	72.1	67.4–76.8	61.2	56.9–65.5	59.8	55.6–64.0	0.86(0.74,0.99)

*Note: by comparing 2015 to 2011

**Fig 1:**
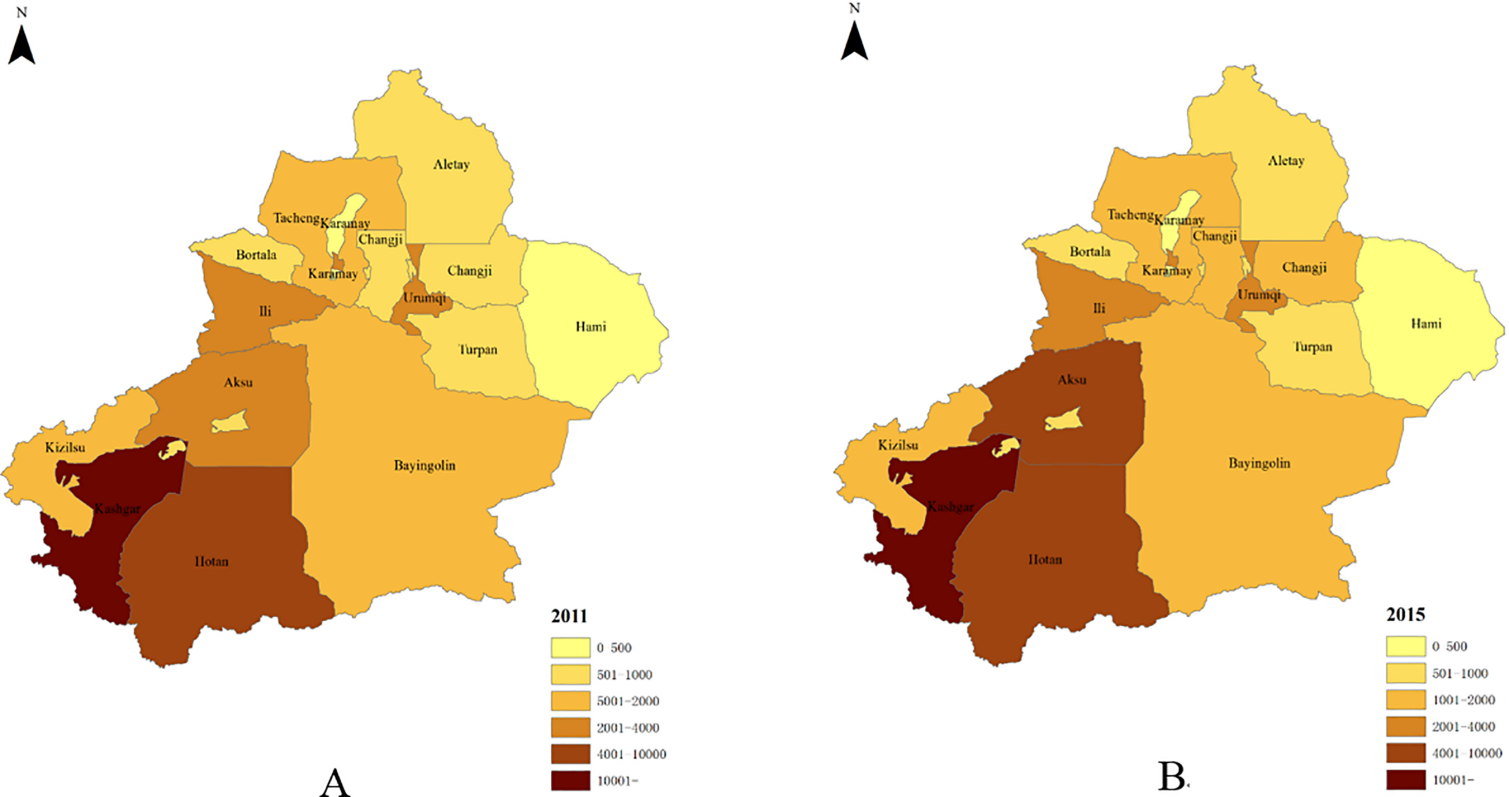
Geological distribution of the number of reported PTB in Xinjiang, China (2011 and 2015). *Note: A is for 2011, and B is for 2015.

The annual incidence of reported PTB among rural residents was much higher than that among urban residents, and the overall reported incidence in the rural population increased from 228.7 cases per 100,000 people (95% CI = 226.0–231.4) in 2011 to 287.8 cases per 100,000 people (95% CI = 284.8–290.8) in 2015. The southern region of Xinjiang Uygur Autonomous Region had the highest reported incidence, while the counties administratively managed by the province had the lowest reported incidence of PTB. In addition, the reported incidence of PTB in the southern region increased from 257.8 cases per 100,000 people (95% CI = 254.7–260.9) in 2011 to 312.7 cases per 100,000 people (95% CI = 309.4–316.0) in 2015 (Table 2). The Northwest region also has a relatively high reported incidence of PTB, specifically in Tacheng and Karamay (Fig 1).

## Discussion

The current study analyzes 212,216 smear-positive PTB cases reported to the TBIMS between 2011 and 2015. We found that the incidence of PTB remains high with an increasing trend. This study contributes to the existing literature by examining the incidence of PTB in one of the highest-burden regions in China [5]. We also identified an increasing trend in reported incidence of cases over time and demonstrated regional disparities in disease burden. Overall our work emphasizes the urgent need for more comprehensive intervention strategies to control PTB in the region, particularly in southern Xinjiang.

Xinjiang has been implementing directly observed treatment, short-course (DOTS) control strategy for more than ten years [16]. However, the present study indicates that the reported incidence of smear positive PTB in Xinjiang remained as high as 195.8 cases per 100,000 people in 2015. Although this reported incidence is lower than the reported incidence from 1990 (231 per 100 000 people) [12], it is higher than the reported regional figure for 2010 (170 per 100,000 people) [6], and is much higher than values reported from eastern and central China [5]. Furthermore, this reported incidence is about 1.6 times the global burden of PTB reported in 2012[17], and higher than many countries in Africa [17, 18]. There are several plausible explanations for such a high reported incidence of PTB in this region. First of all, as one of the most resource-poor regions of China, Xinjiang has a weak primary care system in place [19]. People in Xinjiang, especially rural residents in the Southern region, tend to have poor access to health care. Secondly, Xinjiang, is one of the regions in the country with high reported incidence of multidrug-resistant TB [20]. Unsuccessfully treated cases may exacerbate re-infection in the community and thus result in upsurge and reemergence of PTB. Further, due to socio-political, religious and other logistic barriers, there may be a lack of effective implementation of PTB control programs in this region. Last but not least, the increased PTB incidence may actually be a sign of improved access to health care, especially for people living in the rural areas. As a result of economic development, more TB patients may have gained access to medical facilities, causing TB monitoring systems to pick up more patients and increase the incidence of reported TB. Future studies should aim to confirm these potential reasons and help design targeted intervention strategies.

In 2006, under the supervision of the WHO, Xinjiang implemented the STOP TB strategy [21], which aimed to reduce the reported incidence of death due to TB by 50% between 1990 and 2015. Previous research has shown that the overall reported incidence of smear-positive PTB decreased by 26% from 1990 to 2010–11 [6]. The reported National-level incidence of smear-positive TB also supposedly decreased in all provinces after 2000 [5]. However our results indicate a progressively increase in the reported incidence of smear-positive PTB in the study region and an overall increase in PTB cases of about 10% during the study period (2011–15). This trend was more evident among females, rural residents and those residing in southern region of Xinjiang. In fairness, the observed increase of PTB could be due to improved case detection and reporting. However, this increase raises worrisome questions regarding the effectiveness of ongoing PTB control programs. To curb this upsurge of PTB and achieve the goal of TB elimination by 2030 [17, 21], public health officials might consider reinforcing existing TB control strategies, including DOTS, as well as supplementing targeted intervention strategies, including promotion of TB screening among high-risk population groups.

We observed regional disparity in the reported incidence of PTB, with the highest burden of PTB in the southern region of Xinjiang. This observation was consistent with the results of spatial analysis, which showed “hot spots” of PTB incidence in the Southwest region [22]. Compared to other parts of Xinjiang, the Southern region of Xinjiang is more rural and underdeveloped, with less access to health care; these are all potential factors contributing to higher PTB incidence [23–25]. Another potential contributor to this regional disparity could be the inequity in the allocation of public health resources across Xinjiang: Due to ethnic, social and cultural factors, the Southern region has historically received less attention regarding health care and fewer TB prevention and control programs [26]. To deal with these issues, targeted intervention programs for TB control and elimination should be specifically reinforced in the Southern region of Xinjiang.

We also observed large variations in the reported incidence of PTB between rural and urban populations. Previous studies indicated that rural residents in Xinjiang tend to have less education and access to health care, and reside in the poorer living environments compared to their urban counterparts [27]. PTB patients from rural Xinjiang are likely to present later to the TB treatment clinics, after having a chance to transmit the disease to others [28]. Hence, in order to control PTB in Xinjiang, it is essential to improve primary care capacity and emphasize timely referral and reporting of the TB cases to the DOTS facilities in rural areas.

Our study has several important potential limitations, most of which are related to its surveillance data-based design. First, there may have been duplication of reporting, which could have led to an overestimation of the disease burden. In order to prevent this, study personnel from all county/city level CDC were instructed to double check the cases based on recorded address and other personal information. Second, for each case, only a few socio-demographic (for example, ethnic group) and behavioral variables were recorded in the case report system, limiting the scope of the analysis, especially with regard to risk factors and driving forces behind this epidemic. In addition, these data were not reported individually, which prevented us from conducting traditional trend tests and multivariate logistic regressions. Third, under-reporting due to insufficient access to healthcare systems could be another issue in this current study, and may have resulted in an underestimation of the ongoing epidemic. Finally, we were unable to separate previously existing TB cases from newly diagnosed cases among all reports from 2011 to 2015.

## Conclusion

Overall, the burden of smear-positive PTB was found to be high in Xinjiang, with an increasing trend over time. The southern, rural regions of Xinjiang are experiencing a particularly large increase in reported TB cases, emphasizing the need for targeted intervention strategies.

## Supporting information

S1 File Dataset of the study. (XLS)
